# Structural basis of suppression of host translation termination by Moloney Murine Leukemia Virus

**DOI:** 10.1038/ncomms12070

**Published:** 2016-06-22

**Authors:** Xuhua Tang, Yiping Zhu, Stacey L. Baker, Matthew W. Bowler, Benjamin Jieming Chen, Chen Chen, J. Robert Hogg, Stephen P. Goff, Haiwei Song

**Affiliations:** 1 Institute of Molecular and Cell Biology, 61 Biopolis Drive, Proteos, Singapore 138673, Singapore; 2 Department of Biochemistry and Molecular Biophysics, Columbia University, HHSC 1310C, 701 West 168th Street, New York, New York 10032, USA; 3 Howard Hughes Medical Institute, Columbia University, HHSC 1310C, 701 West 168th Street, New York, NY 10032, USA; 4 Biochemistry and Biophysics Center, National Heart, Lung, and Blood Institute, National Institutes of Health, 50 South Drive, Bethesda, Maryland 20892, USA; 5 European Molecular Biology Laboratory, Grenoble Outstation, 71 Avenue des Martyrs, CS 90181, Grenoble F-38042, France; 6 Unit of Virus Host-Cell Interactions, University Grenoble Alpes-EMBL-CNRS, 71 Avenue des Martyrs, CS 90181, Grenoble F-38042, France; 7 Life Sciences Institute, Zhejiang University, 388 Yuhangtang Road, Hangzhou 310058, China; 8 Department of Biochemistry, National University of Singapore, 14 Science Drive, Singapore 117543, Singapore

## Abstract

Retroviral reverse transcriptase (RT) of Moloney murine leukemia virus (MoMLV) is expressed in the form of a large Gag–Pol precursor protein by suppression of translational termination in which the maximal efficiency of stop codon read-through depends on the interaction between MoMLV RT and peptidyl release factor 1 (eRF1). Here, we report the crystal structure of MoMLV RT in complex with eRF1. The MoMLV RT interacts with the C-terminal domain of eRF1 via its RNase H domain to sterically occlude the binding of peptidyl release factor 3 (eRF3) to eRF1. Promotion of read-through by MoMLV RNase H prevents nonsense-mediated mRNA decay (NMD) of mRNAs. Comparison of our structure with that of HIV RT explains why HIV RT cannot interact with eRF1. Our results provide a mechanistic view of how MoMLV manipulates the host translation termination machinery for the synthesis of its own proteins.

## Abstract (web summary)

Retroviral reverse transcriptase from Moloney Murine Leukemia Virus (MoMLV) requires interaction with peptidyl release factor 1. Here, the authors report the crystal structure of this complex, and provide insights into how MoMLV uses the host translation machinery to synthesize its own proteins.

## Graphical abstract

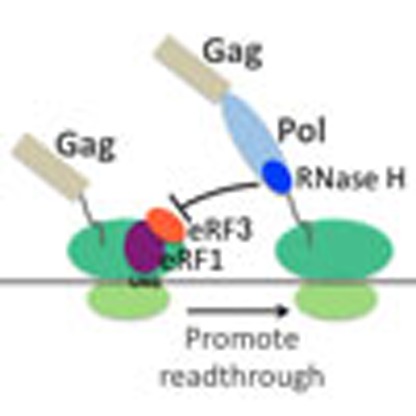

Due to limited genome encoding capacity, viruses are reliant on the host translation system for synthesis of their viral proteins. Various viruses have been found to manipulate almost every step of the host translation process, mainly through targeting cellular translation factors^1^. Most retroviruses utilize translational recoding of a viral messenger RNA (mRNA) stop codon to express the *gag* gene both as an independent polyprotein (Gag) and as a fusion (Gag–Pol) with the polyprotein encoded by the *pol* gene. The ratio of Gag to Gag–Pol is delicately balanced during virus assembly and is critical for infection^2^,^3^. Therefore, retroviruses have developed at least two different strategies to switch between Gag and Gag–Pol expression, both regulated by viral pseudoknots or hairpin RNA structures^4^,^5^,^6^,^7^,^8^. In HIV, the *gag* and *pol* genes are in different reading frames and Gag–Pol protein production requires a −1 ribosomal frameshift at the end of the *gag* gene. However, in Moloney murine leukemia virus (MoMLV), these two genes are in the same reading frame, and the suppression of the *gag* UAG termination codon permits read-through to the *pol* gene^9^. The essential replication enzyme reverse transcriptase (RT) is expressed as part of a Gag–Pol fusion protein, accounting for 5% of unspliced retroviral RNA translation. Biochemical and structural studies showed that the MoMLV RT displays a monomeric architecture containing an N-terminal polymerase domain and a C-terminal RNase H domain (Fig. 1a)^10^. In contrast, the HIV RT is a heterodimer consisting of p66 and p51 subunits^11^, the latter of which is derived by proteolysis of p66 and lacks the RNase H domain.

In eukaryotes, stop codon recognition is initiated by the binding of the essential termination factor eRF1 in complex with a guanosine triphosphate (GTP)-bound eRF3 to the ribosome. The subsequent dissociation of eRF3 after GTP hydrolysis and recruitment of ATP binding cassette E1 (ABCE1) to eRF1 induce peptide release and ribosomal subunit dissociation^12^. Comprehensive structural studies of eRF1 and its complexes with other termination factors have enabled an understanding of how eRF1 coordinates the termination events via its three distinct domains^13^,^14^,^15^,^16^,^17^,^18^, namely the N-terminal domain (eRF1-N), M-domain (eRF1-M) and C-terminal domain (eRF1-C) (Fig. 1a). eRF1-N (aa 1–142) contains a conserved Asn-Ile-Lys-Ser (NIKS) motif and has an essential role in stop codon recognition^13^. eRF1-M (aa 143–276) mimics the transfer RNA (tRNA) acceptor stem and harbours a universal GGQ tip to stretch toward the CCA end of the peptidyl tRNA for triggering peptide release^13^,^17^. eRF1-C (aa 277–437) functions to recruit other termination factors such as eRF3 when forming the pre-termination complex^13^,^14^,^16^.

Previously, we showed that MoMLV utilized its RT to interact with host eRF1, thereby promoting stop codon read-through to make Gag–Pol^19^. However, the molecular events underlying this mechanism remain undefined. In this study, we determine the crystal structure of MoMLV RT in complex with the full-length mouse eRF1. Our structure shows that the MoMLV RT interacts with the C-terminal domain of eRF1 via its RNase H domain. Structure-guided functional assays suggest that MoMLV RT suppresses translation termination by outcompeting eRF3 for binding to eRF1. We also show that MoMLV RNase H promotes stop codon read-through, which in turn prevents nonsense-mediated mRNA decay (NMD). Altogether, these results reveal the structural basis of host translation termination suppression by MoMLV.

## Results

### Structural determination

**Figure 1:**
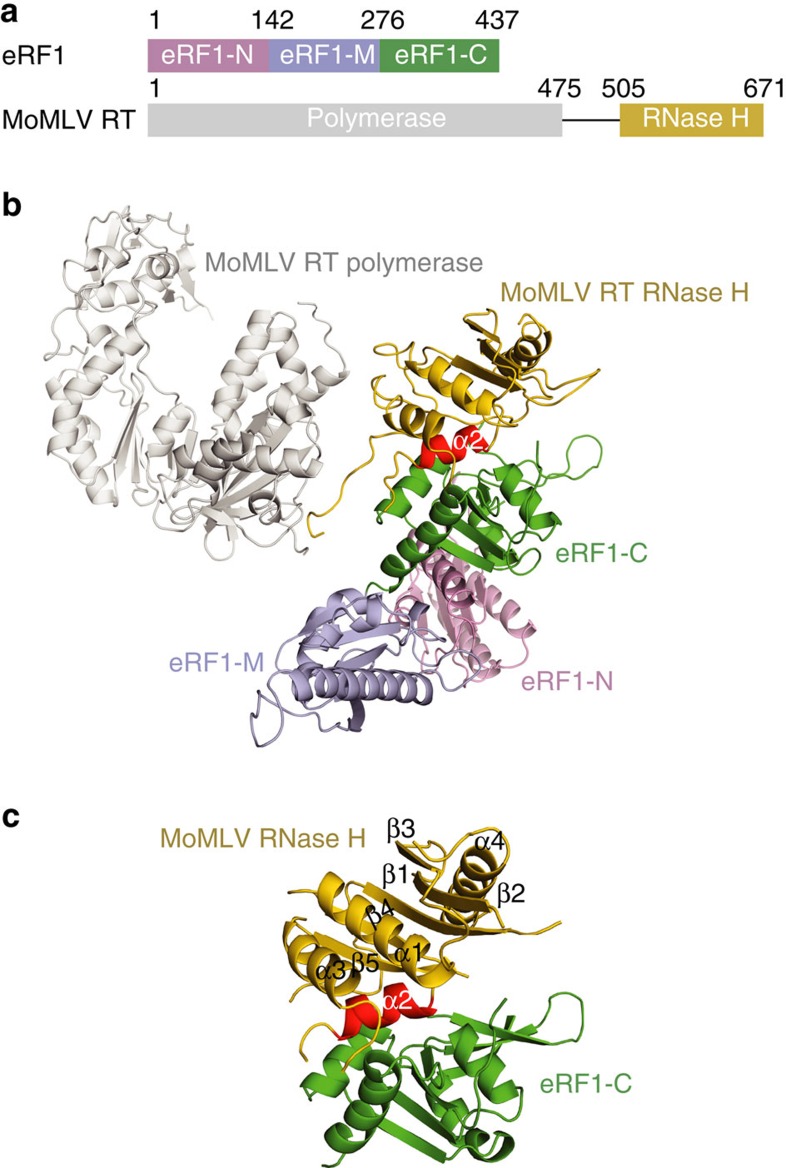
Structure of the MoMLV RT/eRF1 complex. (**a**) Schematic representation of the domain organization of eRF1 and MoMLV RT. Domains N, M, and C of eRF1 are coloured in pink, lightblue and green, respectively. MoMLV RT polymerase domain is coloured in grey and RNase H domain in yellow. (**b**) A ribbon diagram of the MoMLV RT/eRF1 complex. The colouring scheme is as in **a**. The helix α2 of RNase H domain is highlighted in red. (**c**) The RNase H/eRF1-C complex structure. The secondary structure elements of RNase H domain are labelled.

We solved the crystal structure of MoMLV RT (aa 24–671) in complex with the full-length mouse eRF1 at a resolution of 4.0 Å (for simplicity designated as MoMLV RT/eRF1) (Fig. 1b). MoMLV RT contains an N-terminal polymerase domain and C-terminal RNase H domain connected by a flexible linker, which is not visible in the electron density map and is assumed to be disordered. Structural comparison of the RT polymerase domain in our structure with that in a MoMLV RT/DNA complex^20^ and a Xenotropic murine leukemia virus-related virus (XMRV) RT/RNA/DNA ternary complex^10^, reveals that the binding of eRF1 did not cause a significant conformational change in MoMLV RT's polymerase domain (Supplementary Fig. 1a). The catalytic residues (D524, E562, D583 and D653) of RNase H are distant from the bound eRF1 (Supplementary Fig. 1b). The RNase H activity assays further confirmed that eRF1 binding does not affect the RNase H activity (Supplementary Fig. 1c, upper panel). These findings are consistent with our previous data that the MoMLV RT–eRF1 interaction does not affect RT's activities^19^.

The MoMLV RT–eRF1 interaction is mediated via the contacts of the RNase H domain of MoMLV RT with the C-terminal domain of eRF1. To verify this interaction, we also determined the crystal structure of the isolated RNase H domain of MoMLV RT in complex with eRF1-C at a resolution of 2.8 Å (Fig. 1c). As the RNase H/eRF1-C interface is identical with that observed in the MoMLV RT/eRF1 complex, subsequent analysis concerning the RT–eRF1 interaction only refers to the RNase H/eRF1-C structure.

### Interaction of MoMLV RT with eRF1

**Figure 2:**
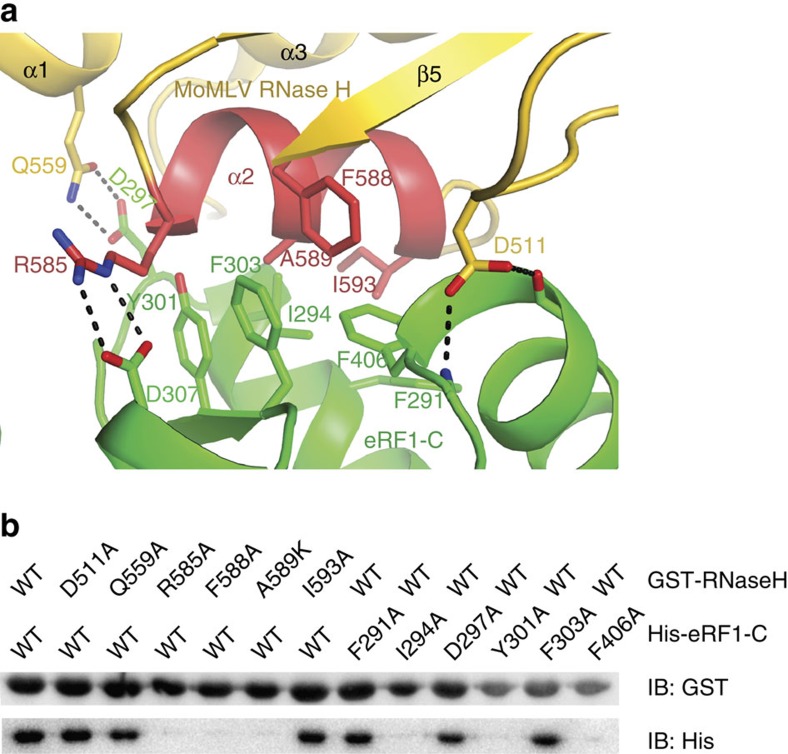
Interaction of MoMLV RT with eRF1. (**a**) Interface between the RNase H domain of MoMLV RT and eRF1-C. Residues involved in the interaction are shown as sticks and labelled. (**b**) GST pull-down assay. GST tagged WT MoMLV RNase H and its mutants on beads were used to bind His-tagged eRF1-C and its variants.

The interaction of the RNase H domain of MoMLV RT with eRF1-C buries a total solvent accessible surface area of 1173 Å^2^. The RNase H domain mainly uses its helix α2 to interact with eRF1-C through predominantly hydrophobic contacts (Fig. 2a). Residues Phe588, Ala589, Ile593 and the methylene group of Arg585 in helix α2 of the RNase H domain form a hydrophobic patch that interacts with the hydrophobic residues of eRF1-C, including Phe291 and Ile294 in helix α8, Tyr301 and Phe303 in the loop region connecting α8-α9 and Phe406 in loop α11-β11 (numbering as previously described^16^). In addition to these predominant hydrophobic interactions, Arg585 of the RNase H domain forms a salt bridge with Asp307 of eRF1-C, Gln559 of RNase H is hydrogen bonded to Asp297 of eRF1-C, and Asp511 of RNase H forms hydrogen bonds with the carbonyl groups of Lys404 and Gly407 in helix α11 of eRF1-C.

To examine the role of the RNase H/eRF1-C interface, we mutated several residues and examined their effects on binding by glutathione S-transferase (GST) pull-down assays (Fig. 2b). Mutation of Ile294, Tyr301 or Phe406 of eRF1-C to alanine disrupted its interaction with RNase H. Likewise, single mutations in the isolated RNase H domain (R585A, F588A and A589K) of MoMLV RT abolished its binding to eRF1-C (Fig. 2b). Residues Ile294, Tyr301, Phe406 of eRF1-C and Phe588 of RNase H are the key components of the hydrophobic RNase H/eRF1-C interface. Single Ala mutations of these residues disrupted the RNase H/eRF1-C interaction. Mutation of Arg585 to Ala would abolish its salt bridge interaction with Asp307, while substitution of Ala589 by a lysine would place a charged residue inside the hydrophobic interface, thereby destabilizing the RNase H/eRF1-C interaction. In contrast, single Ala substitutions of Asp511, Gln559 and Ile593 of RNase H and Phe291, Asp297 and Phe303 of eRF1 had no effect on the RNase H/eRF1-C interaction. Residues Asp511, Ile593 and Qln559 of RNase H, and Phe291 and Asp297 of eRF1 are located at the edge of the main interface and would not be predicted to contribute significantly to the RNase H/eRF1-C interaction, and therefore it is not surprising that mutations of these residues had no effect on the interaction. Phe303 of eRF1 is situated in the middle of the interface and appears to be a key hydrophobic residue. One plausible explanation for the F303A mutant of eRF1 lacking the RNase H binding defect is that the side chain of Arg585 could rotate and place its methylene group in a position to compensate for the loss of the phenol group of F303A mutant.

We further examined the binding thermodynamics of wild-type (WT) RNase H and its variants to eRF1 by isothermal titration calorimetry (ITC). WT RNase H binds to eRF1 with an equilibrium dissociation constant (*K*_d_) of ∼4.18 μM. The RNase H mutant F588A showed ∼10-fold reduced binding to eRF1, whereas the binding of R585A or A589K to eRF1 was abrogated (Supplementary Fig. 2a–d). These data indicate that helix α2 of the RNase H domain in MoMLV RT harbouring these residues is critical for the RT–eRF1 interaction.

We note that earlier mutational studies found that mutation G525E led to a strong disruption of RT–eRF1 binding as assessed in a yeast two-hybrid assay^19^. Gly525 is far from the RT–eRF1 interface and is located in the interior of the RNase H domain. Its substitution by a glutamate acid places a negatively charged residue with a long side chain inside the hydrophobic core of the RNase H domain, therefore destabilizing the fold of RNase H. Thus, we suggest this mutation acts indirectly.

The interaction of polymerase domain of RT with eRF1 by GST pull-down assay was only detectable in low salt condition in the previous study^19^. Thus, the crystallization buffer conditions in this study may prevent the polymerase domain of RT from interacting with eRF1. Intriguingly, we observe an interface between the RT polymerase domain and eRF1 within the crystal lattice in our MoMLV RT/eRF1 structure, but only between symmetry-related molecules. We postulate that this observation may only be due to the crystal packing, because the electron density of this interface is not well defined, indicating a certain degree of disorder in this area.

### The RT–eRF1 interaction enhances read-through

**Figure 3:**
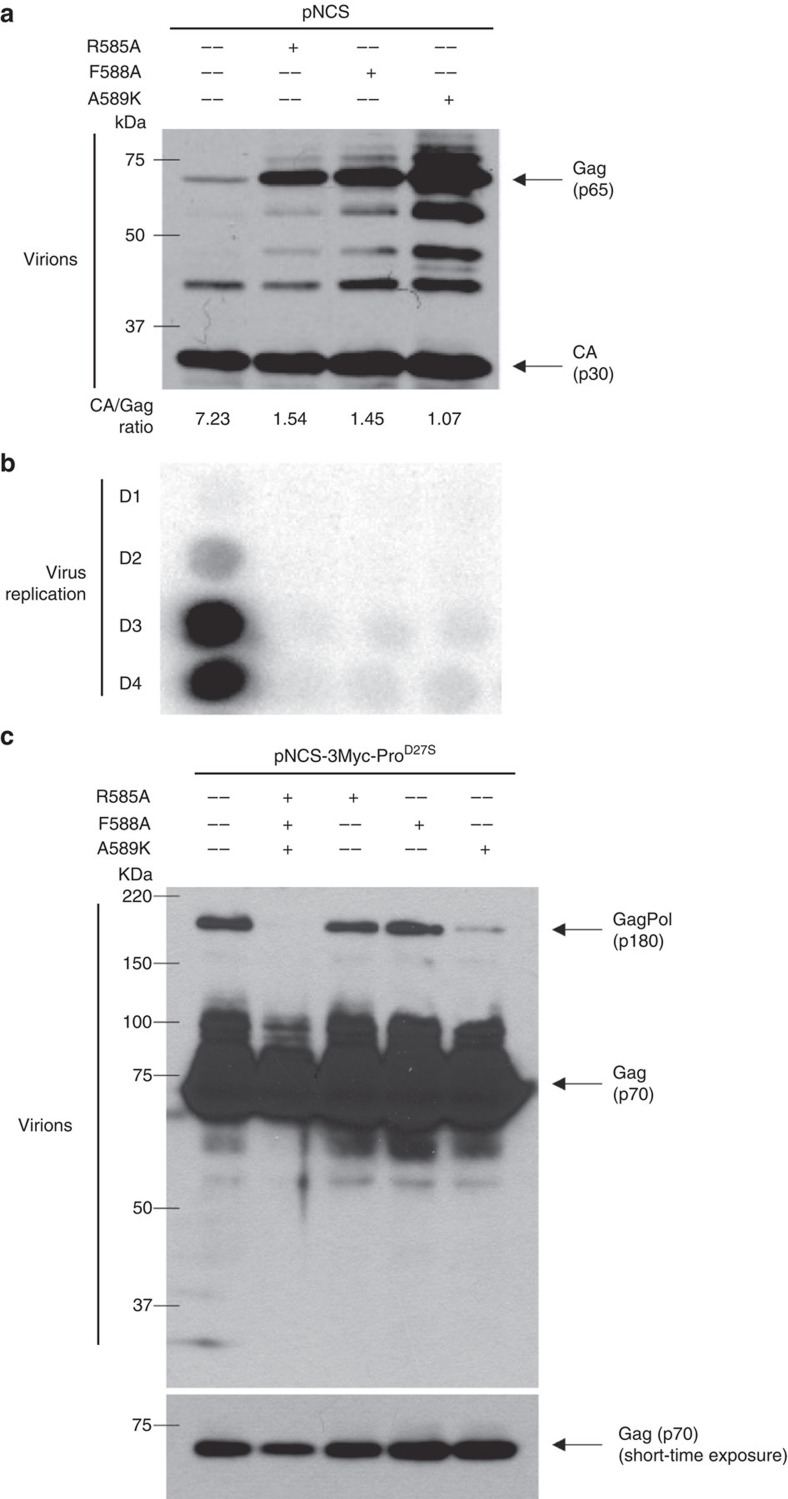
Non-interacting RT mutants showing reduced read-through are replication defective. (**a**) The provirus carrying mutants that are unable to bind eRF1 show reduced read-through efficiency. The proviral DNA were used to transform 293 T cells and the purified virions were analyzed by western blot. The virion produced by WT DNA contained high levels of capsid (CA, ∼30 kDa), while mutants showed poor Gag (∼65 KDa) cleavage as a consequence of low read-through. (**b**) Non-interacting RT mutants are replication defective. Release of spreading virus was detected on the indicated day post infection. (**c**) HEK 293 T cells were transfected with WT pNCS-3Myc-Pro^D27S^ or pNCS-3Myc-Pro^D27S^ bearing indicated mutations. Forty-eight hour after transfection, the viruses in the supernatant were collected and purified by ultra-centrifuge through the 25% sucrose cushion. The virus pellets were re-suspended in protein loading buffer, resolved in SDS–PAGE, and visualized by western blot using c-Myc antibody (9E10; sc-40, Santa Cruz; 1:1000 dilution). Noted that the Gag protein has a molecular mass of ∼70 kDa as carrying the 3Myc tag.

To determine the effect of the MoMLV RT–eRF1 interaction on translational read-through, we introduced the RT mutations into the MoMLV provirus and examined the expression levels of the Gag precursor protein and capsid protein (CA) in virion lysates. The read-through efficiency is evaluated here by examination of the CA/Gag ratio, as the processing of Gag precursor protein to CA protein is mediated by PR protease, which depends on read-through for its expression. Compared with the WT virions, those with mutations affecting eRF1 binding (R585A, F588A or A589K) showed dramatically increased ratios of Gag precursor to mature CA protein, indicating that a large fraction of the Gag precursor proteins remained unprocessed (Fig. 3a). Although we cannot rule out other mechanisms for the effects of the RT mutations, the results are consistent with a reduced expression of read-through-dependent PR protease present in the Gag–Pol precursor. The viruses were then used to infect Rat2 cells to detect their ability to spread. Although WT viruses rapidly spread in the cultures, all mutants were replication defective (Fig. 3b). This finding is consistent with the notion that the interaction with eRF1 is important for replication, as these mutations have little or no effect on their RNase H activities (Supplementary Fig. 1c, lower panel). To further examine the role of the RT–eRF1 interaction in read-through, selected mutations (R585A, F588A or A589K) were introduced into an MoMLV provirus in which the protease catalytic site was mutated (Pro^D27S^). In this context, the uncleaved Gag–Pol precursor with WT RT was readily detected in virion particles, while the Gag–Pol protein with triple mutations (R585A/F588A/A589K) was undetectable. The A589K mutation significantly reduced Gag–Pol levels, while the R585A or F588A mutations had no effect on Gag–Pol levels (Fig. 3c). We conclude that A589 is the most important amino acid for RT-mediated enhancement of read-through. These observations support the hypothesis that the read-through-enhancing interaction of RT with eRF1 is required for viral replication.

### MoMLV RT outcompetes eRF3 for binding to eRF1

**Figure 4:**
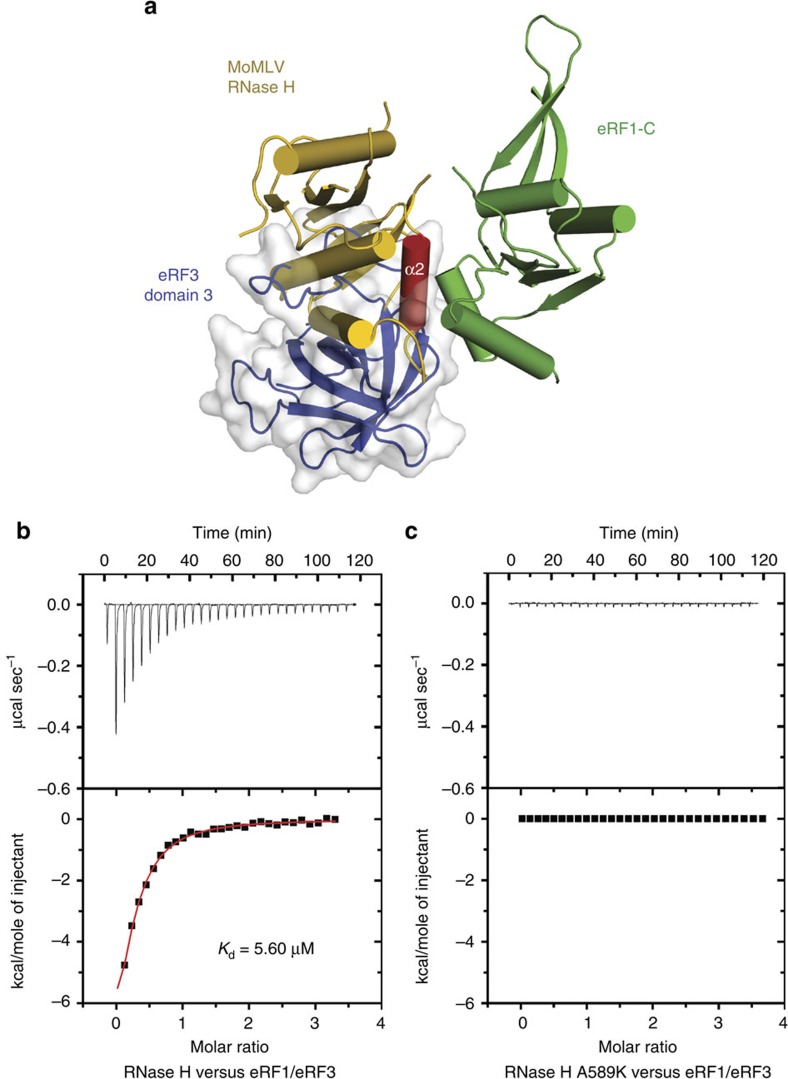
The RNase H domain of MoMLV RT outcompetes eRF3 for binding to eRF1. (**a**) Superposition of the RNase H/eRF1-C complex with the eRF1/eRF3 complex (PDB accession code: 3E1Y) at eRF1-C domain. The overlapping interface suggests that MoMLV RT and eRF3 are mutually exclusive for binding to eRF1. eRF3 domain 3 is shown in cartoon (blue) covered with grey transparent surface. eRF3 domain 2 and eRF1-C domain in eRF1/eRF3 complex are not displayed for clarity. (**b**,**c**) Representative ITC titrations of WT RNase H and mutant A589K to the eRF1/eRF3 complex. The upper panels show the binding isotherms and the lower panels show the integrated heat for each injection fitted to a single-site model.

eRF1 and eRF3 interact predominantly via their respective C-terminal domains (eRF1-C and domain 3 of eRF3) and bind to the ribosomal pre-termination complex as a stable eRF1/eRF3 complex for stop codon decoding^15^,^16^. Structural superposition of MoMLV RT/eRF1 and eRF1/eRF3 complexes showed that eRF1 interacts with both MoMLV RT and eRF3 through overlapping surface regions (Fig. 4a), suggesting that MoMLV RT and eRF3 are mutually exclusive for binding to eRF1. In support of this structural observation, WT RNase H but not mutants defective in eRF1 binding efficiently outcompetes eRF3 for binding eRF1 with an apparent *K*_d_ of ∼5.6 μM, despite eRF3 exhibiting at least 10 times higher affinity for eRF1 than RNase H (Fig. 4b,c; Supplementary Fig. 2e–g). These results suggest that MoMLV RT likely suppresses translation termination by outcompeting eRF3 for binding to eRF1.

### Promotion of read-through by RNase H prevents NMD

**Figure 5:**
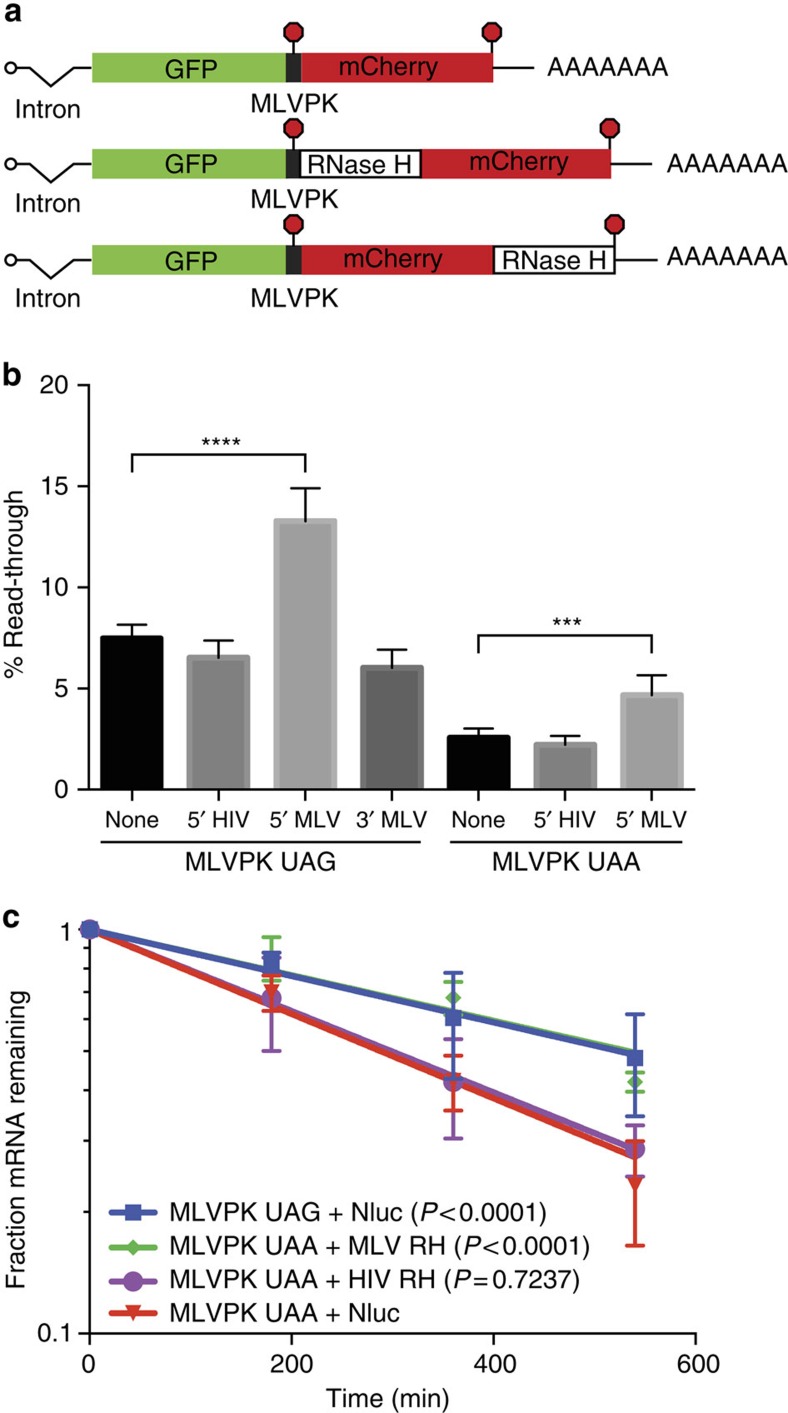
MoMLV RNase H enhances stop codon read-through and stabilizes mRNAs. (**a**) Schematic of tet-regulated reporter mRNAs used in read-through and mRNA decay assays. The indicated RNase H variants were inserted downstream of the MLVPK, in-frame with the GFP and mCherry ORFs. The red dot represents the stop codon. (**b**) Translational read-through assays using dual-fluorescent-protein reporters containing the indicated MLVPK and RNase H variants. The ratio of mCherry:GFP from each experimental construct was normalized to that arising from a sequence-matched control lacking a stop codon between GFP and mCherry. Error bars indicate s.d. (*n*=7; ****P<0.0001; ***P<0.001 in one-way ANOVA with Sidak's multiple comparisons test). (**c**) Semi-log plot of levels of the indicated tet-regulated mRNAs throughout pulse-chase mRNA decay assays. Best-fit lines calculated by the least-squares method are indicated; error bars denote s.d. (*n*=4; *P* values were calculated using ANCOVA analysis, comparing the indicated mRNAs to MLVPK UAA+Nluc).

We introduced MoMLV or HIV RNase H immediately downstream of the MoMLV pseudoknot (MLVPK) in dual-fluorescent-protein reporters of translational read-through (Fig. 5a). These reporters mimic the natural context of RNase H expression, and could either allow activity in *trans* (on different mRNAs following termination and release) or in *cis* (at the upstream termination codon on the same mRNA). In the latter scenario, termination suppression activity may be enhanced by tethering of newly synthesized RNAse H via the nascent polypeptide. This positioning of the MoMLV RNase H domain led to ∼twofold more read-through than control constructs containing mCherry alone or the HIV RNase H and mCherry downstream of the MoMLV pseudoknot. Stimulation of read-through by MoMLV RNase H was evident when using both the WT UAG stop codon and a UAA stop codon, which reduces read-through to ∼2% in controls. When moved to a position 3′ of the mCherry open reading frame (ORF), the MoMLV RNase H failed to stimulate read-through. Because polypeptides containing C-terminal RNase H would undergo termination and release immediately after completion of RNase H synthesis, this finding suggests that nascent polypeptide-mediated tethering of MoMLV RNase H to the translating mRNA is important for optimal function (Fig. 5b).

Termination at the MoMLV *gag* stop codon results in an mRNA with an effective 3′UTR length of ∼6,000 nt, a feature expected to make the transcript susceptible to the conserved NMD pathway^21^ in the absence of a protective mechanism. As previous observations show that induction of read-through by the MoMLV pseudoknot can potently inhibit NMD of reporter transcripts^22^, we investigated the ability of MoMLV RNase H to augment this activity in pulse-chase decay assays of tetracycline-regulated dual-fluorescent-protein reporters. Because degradation by NMD is strongly 3′UTR length-dependent, we used either HIV RNase H or NanoLuc luciferase (Nluc) to equalize 3′UTR length among the constructs used. Read-through stimulated by the WT MoMLV pseudoknot (UAG) was sufficient to fully stabilize mRNAs with the extended mCherry-Nluc 3′UTR, likely due to efficient displacement of the NMD key effector Upf1 from the 3′UTR by elongating ribosomes^22^ (half-life calculated from best-fit line=519 min, 95% confidence of interval (CI)=423–670 min). Therefore, to test a potential role for the RNase H domain in contributing to NMD inhibition, we used the impaired UAA MoMLV pseudoknot variant, which promoted partial rescue of mRNAs containing Nluc or HIV RNase H 3′UTR extensions (Nluc: best-fit=286 min, 95% CI=256–322 min; HIV RNase H: best-fit=296 min, 95% CI=251–360 min). Consistent with the read-through data above, the presence of the MoMLV RNase H dramatically increased RNA stability, resulting in a half-life of ∼530 min (95% CI=457–653 min). This half-life is similar to that exhibited by mRNAs containing the UAG pseudoknot and Nluc, consistent with a model in which promotion of read-through by MoMLV RNase H in turn prevents NMD (Fig. 5c).

## Discussion

Translation termination and ribosome recycling are two critical processes in protein biosynthesis. During translation termination, a stop codon in the ribosomal A site is decoded by eRF1 delivered by eRF3 in the form of an eRF1/eRF3/GTP ternary complex. After GTP is hydrolyzed, eRF3 dissociates from the ribosome, and ABCE1 binds to the eRF1-bound ribosome to stimulate peptide release and subsequent ribosomal subunit dissociation^23^,^24^. Structural analysis showed that eRF1 uses its C-terminal domain to interact with both eRF3 and ABCE1 (refs 13, 16). Our structural and functional studies of the MoMLV RT/eRF1 complex showed that MoMLV RT likely suppresses stop codon read-through via outcompeting eRF3 binding to eRF1. Since the same surface of eRF1 is used for binding both eRF3 and ABCE1, MoMLV RT would be predicted to compete with ABCE1 for binding to eRF1, therefore inhibiting peptide release and possibly ribosomal subunit dissociation as well. In this scenario, the interval between eRF3 GTP hydrolysis/dissociation and ABCE1 binding would give MoMLV RT a window of time to interact with and inhibit eRF1.

Retroviruses appear to have evolved multiple mechanisms to protect their mRNAs from NMD. Recent data show that a *cis*-acting RNA stability element immediately downstream of *gag* in unspliced Rous sarcoma virus (RSV) RNA recruits polypyrimidine tract binding protein 1 to protect its long 3′UTR from NMD^25^. In deltaretrovirus human T-lymphotropic virus type 1, it was observed that the viral protein Tax inhibits mRNA degradation via interacting with INT6 and preventing the association of INT6 with Upf1 (ref. 26). A possible alternative retroviral mechanism for NMD escape was suggested by findings that insertion of the read-through-promoting MLVPK sequence into NMD reporter transcripts antagonizes Upf1 recruitment and subsequent mRNA decay^22^. In addition to promoting stability by enhancing translational read-through, MoMLV RNase H may have a more direct role in protecting viral mRNAs from decay. Upf1 interacts with the C-terminal domain of eRF1 and the GTPase domain of eRF3 to trigger NMD^27^,^28^. Our structure shows that MoMLV RT interacts with eRF1 to occlude eRF3 and suppress termination. Therefore, the MoMLV RT–eRF1 interaction may prevent the recruitment of Upf1 to the terminating ribosome by disrupting the Upf1–eRF1 interaction, thereby allowing the *gag-pol* mRNA to bypass degradation by NMD.

**Figure 6:**
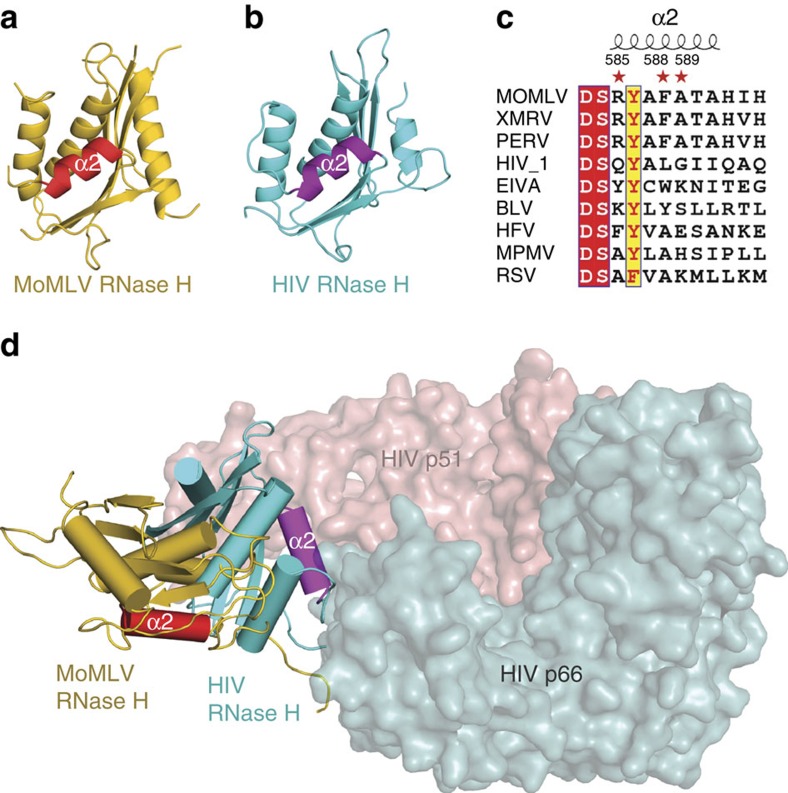
Structural explanation of why HIV RT cannot interact with eRF1. (**a**) Cartoon representation of the RNase H domain of MoMLV RT. (**b**) The RNase H domain of HIV RT, which is coloured in cyan with its helix α2 highlighted in purple. (**c**) Sequence alignment of the helix α2 from various genera of retroviruses. MoMLV, XMRV and PERV (Porcine endogenous retrovirus) belong to gammaretrovirus. HIV-1 and EIVA (Equine infectious anaemia) are lentivirus. BLV (Bovine leukemia virus), HFV (Human foamy virus), MPMV (Mason pfizer monkey virus) and RSV (Rous sarcoma virus) belong to deltaretrovirus, spumaretrovirus, betaretrovirus and alpharetrovirus respectively. (**d**) Superposition of MoMLV RT and HIV RT p66 (PDB accession code: 1HYS) at their polymerase domains. HIV RT p66 except RNase H domain and p51 subunits are shown as surfaces coloured in cyan and salmon, respectively.

The RNase H domain is structurally well conserved among retroviral RTs and exhibits similar substrate recognition activity (Fig. 6a,b). Sequence alignment of helix α2 in RNase H domains from various retroviruses reveals that the three eRF1-interacting amino acids of MoMLV RT—R585, F588 and A589—are identical in gammaretroviruses, which utilize read-through for Gag–Pol production but are highly variable in other genera of retroviruses that utilize frameshifting (Fig. 6c; Supplementary Fig. 3a). The residues equivalent to both R585 and F588 are replaced by an alanine in alpharetrovirus RSV and betaretrovirus Mason pfizer monkey virus (MPMV), while the residues equivalent to A589 are substituted by a lysine in RSV, a histidine in MPMV and a glutamate in spumaretrovirus human foamy virus. This observation and our ITC data described above suggest that R585, F588 and A589 in MoMLV RT are structural determinants for its interaction with eRF1, and alterations of these key residues in other genera of retroviruses would render their RTs being unable to bind to eRF1.

The conformational flexibility of the retroviral RNase H domain, which represents the major difference between the monomeric and dimeric retroviral RTs^10^, may also account for the species-dependent specificity of the MoMLV RT–eRF1 interaction. The RNase H domain in monomeric RTs is highly flexible and disordered in previously solved monomeric RT structures including MoMLV RT in complex with dsDNA^20^ and XMRV RT in complex with a DNA/RNA hybrid^10^. Our MoMLV RT/eRF1 structure reveals the full-length monomeric RT architecture for the first time, made possible by the fact that the orientation of the RNase H domain is fixed upon binding eRF1. Structural superposition of MoMLV and HIV RTs based on the polymerase domain showed that MoMLV RNase H swings 92° to a completely different position, accompanied by a moderate movement of thumb and connection subdomains (Fig. 6d). This movement is conferred by a 32 amino acid linker region between the connection and RNase H domains of MoMLV RT, whereas this loop region is not found in HIV RT and is relatively short in other genera of retroviruses (Supplementary Fig. 3b). Moreover, the RNase H domain of HIV RT is spatially locked by the p51 subunit, and consequently its helix α2 is not accessible for binding eRF1 (Fig. 6d; Supplementary Fig. 3c). The presence of a long flexible linker that is essential for MoMLV viral replication^29^, together with the lack of a p51-like subunit to restrict RNase H movement, accounts for the wide-range of RNase H motion observed in MoMLV RT. Taken together, these data suggest that translation termination suppression mediated by the RT–eRF1 interaction appears to represent a unique tactic used by gammaretroviruses, typified by MoMLV.

The expression levels of Gag and Gag–Pol in most retroviruses are delicately balanced to maintain at a ratio close to 20:1, which is critical for infection^2^. The production of Gag–Pol results from a translational recoding event, which involves either ribosomal frameshifting or read-through of the *gag* stop codon^30^. In MoMLV, although a RNA pseudoknot structure downstream of the gag stop codon directs the read-through process^6^,^31^, the maximal efficiency of read-through also requires the MoMLV RT/eRF1 interaction^19^. The binding of eRF1 by MoMLV RT upregulates read-through, thereby creating a positive feedback loop to drive the synthesis of more Gag–Pol. The effect in the context of the full-length viral mRNA may be strongest in *cis*, with the nascent Gag–Pol precursor reaching back to interact with eRF1, thereby enhancing read-through for the next ribosome coming along (Supplementary Fig. 4). The outcompetition of eRF3 by MoMLV RT for eRF1 binding would be expected to require a high local concentration of MoMLV RT, as the binding affinity of MoMLV RT to eRF1 is much weaker than that of eRF3 to eRF1. The ultimate determinants of the frequency of read-through will lie in the binding equilibriums for all the interactions involving the binding of the RNA pseudoknot, RT, eRF1/eRF3, mRNA as well as ABCE1 to the terminating ribosome in such a feedback loop.

## Methods

### Protein expression and purification

Full-length mouse eRF1 (sharing 100% sequence identity with human eRF1) and its N-, M- and C-domains, as well as MoMLV RT lacking its N-terminal region (aa 24–671), an isolated RT polymerase domain (aa 24–499) and an isolated RT RNase H domain (aa 500–671) were cloned into the vector pGEX-6P-1 (GE Healthcare) and expressed as GST fusion proteins in *E. coli* BL21-CodonPlus(DE3)-RIL strain (Agilent Technologies). The mutants were created using QuikChange II XL Site-Directed Mutagenesis Kit (Agilent Technologies). WT and mutant proteins were purified by Glutathione Sepharose 4B resin, followed by PreScission protease cleavage, ion exchange and gel filtration chromatography (GE Healthcare). To make the RT/eRF1 complex, equal molarity of RT and eRF1 were mixed in gel filtration buffer (20 mM HEPES pH 7.5, 150 mM NaCl and 2 mM DTT) and loaded into the Hiload Superdex 200 column (GE Healthcare). The fractions containing the complex were pooled and concentrated to around 10 mg ml^−1^. The RNase H/eRF1-C complex was similarly prepared but purified instead using the Hiload superdex 75 column (GE healthcare) equilibrated with gel filtration buffer (20 mM HEPES pH 7.5, 150 mM NaCl and 5 mM DTT) and concentrated to 65 mg ml^−1^.

### Crystallization and structure determination

The MoMLV RT/eRF1 complex crystals were obtained in the crystallization condition containing 0.02 M magnesium chloride, 0.1 M HEPES pH 7.5, 20% polyacrylic acid sodium salt 5100 by the hanging-drop vapour diffusion method at 4 °C. The crystals were cyroprotected by the mother liquor supplemented with 25% (v/v) ethylene glycol before flash freezing in liquid nitrogen. The RNase H/eRF1-C complex crystals were grown by mixing 1 μl protein with 1 μl reservoir solution containing 0.1 M Tris pH 8.5 and 18% ammonium dihydrogen phosphate at 15 °C and 25% (v/v) glycerol supplemented reservoir condition was used as cryo-protectant. The X-ray diffraction data of the MoMLV RT/eRF1 complex crystals were collected on the beamline ID-23-1 (ESRF, Grenoble, France) and beamline PX-I (SLS, PSI, Switzerland). The diffraction images of the RNase H/eRF1-C complex crystals were collected on beamline I02 (DLS, UK and the beamline BL13B1 (NSRRC, Taiwan)). The data were processed by the XDS programme^32^. Both structures were solved by molecular replacement with PHASER^33^. The models were further manually built using COOT^34^, refined by CNS^35^ using the DEN methodology^36^, Phenix^37^ and REFMAC5 (ref. 38). Structure validation was performed using PROCHECK^39^. The statistics for the diffraction data and structure refinement are listed in Table 1. Stereo image of a representative part of electron density map around the RNase H helix α2 is shown in Supplementary Fig. 5.

### *In vitro* binding experiments

Overall, 50 μl beads bound with WT GST-tagged MoMLV RNase H and its mutants were incubated with 1 μg of purified His-tagged eRF1-C protein at 4 °C for 1 h in buffer containing 20 mM HEPES pH 7.5 and 150 mM NaCl. The beads were washed several times and the bound proteins were analyzed by SDS–PAGE gel and western blotting.

### Isothermal titration calorimetry

ITC measurements were performed at 22 °C using MicroCal VP-ITC (MicroCal Inc.). Protein samples were dialyzed into a buffer containing 20 mM HEPES pH 7.5, 150 mM NaCl and 5 mM TCEP. A sample syringe with stirring speed of 290 r.p.m. was used to titrate the injectant protein (220–300 μM) into a cell containing 13–20 μM protein. The titration comprised 29 injections of 10 μl each, separated by 240 s equilibration time. The datasets were analyzed using the Origin 7.0 program, fitted to a single-site binding model.

### RNase H activity assays

RNase H activity assays were carried out as previously described^40^. A RNA template (5′ UCUUUUCAACGACGAAAAGA 3′) was heat annealed to a threefold molar excess of DNA primer (5′ TCTTTTCGTTG 3′) to yield a RNA–DNA duplex substrate. MoMLV RNase H was incubated with RNA–DNA duplex at 37 °C in buffer containing 50 mM Tris (pH 8.0), 60 mM KCl, 0.1 mg ml^−1^ bovine serum albumin, 2 mM dithiothreitol and 0.5 mM EDTA. Reactions were initiated by the addition of 0.5 mM MnCl_2_ and were stopped at 10 min by adding an equal volume of 100% formamide containing bromophenol blue. RNase H cleavage products were resolved on 15% polyacrylamide—7 M urea gels and stained by GelRed (Biotium).

### Virus purification and virus replication assays

WT pNCA was described previously^19^. Lenti-X 293 T (Clontech) and Rat2 cells were maintained in DMEM medium supplemented with 10% FBS. For virus packaging, Lenti-X 293 T cells in 60 mm dishes were transfected with 5 μg plasmids using Lipofectamine LTX with Plus Reagent (Life Technologies). Two days after transfection, cells were lysed for western blot to analyze the expression of MoMLV proteins. The supernatant of transfected cells was filtered through 0.45 mm membrane and further ultra-centrifuged through the 25% sucrose cushion at 35,000 r.p.m. for 2 h to purify the virus. For replication assay, Rat2 cells in 6-well plate were infected with 100 ml supernatants from MoMLV provirus DNA (WT or mutants) transfected 293 T cells for 3 h. The supernatants were taken out everyday after infection to detect the reverse transcriptase activities. pNCS-3Myc-Pro^D27S^ was constructed from pNCS-3Myc by mutating the aspartate in the protease catalytic site to serine to eliminate the protease activity. pNCS-3Myc was provided by Dr Eran Bacharach (Tel Aviv University, Tel Aviv, Israel), which contains three myc tag repeats in the p12 domain of Gag as described previously^41^.

### Read-through and mRNA decay assays

To create vectors for read-through and mRNA decay assays, the EGFP and mCherry were cloned into the tetracycline-regulated pcTET2 βwtβ vector^42^, replacing all β-globin sequence. The hybrid intron from the pCI vector (Promega) was amplified by PCR and inserted into the HindIII site of the resulting vector to create pcTET2iFP. The indicated MLVPK variants were inserted between the GFP and mCherry ORFs, placing the stop codon in-frame. Control constructs in which the stop codon was replaced by a CAG codon were generated for each sequence variant. Codon-optimized RNase H domains from HIV-1 or MoMLV containing the catalytic D524N mutation or Nano luciferase (Promega) were cloned in-frame downstream of MLVPK or mCherry as indicated.

For dual-fluorescent-protein read-through assays, 293 Tet-off cells (Clontech) were maintained in DMEM supplemented with 10% FBS, and transfected with Turbofect (ThermoFisher) according to the manufacturer's instructions. Cells were collected 48 h post transfection in 1 × passive lysis buffer (Promega), and fluorescence was measured on a Tecan Infinite F200 plate reader. Read-through efficiency was determined by normalizing the ratio of GFP:mCherry fluorescence from the experimental construct to GFP:mCherry fluorescence from a sequence-matched control plasmid lacking a termination codon between GFP and mCherry, as previously described^43^.

mRNA decay assays were performed using Hela Tet-off cells (Clontech) as previously described^22^ with minor modifications. Cells were split to 60 mM plates at a density of 5 × 10^5 ^cells per plate and Turbofect (ThermoFisher) was used for transfection with 800 ng of the indicated experimental vector, 200 ng of pcDNA GFP TP^22^ as a co-transfection control, and 500 ng pcDNA 3.1 empty vector, in the presence of 2 ng ml^−1^ doxycycline. Following a 24-h incubation, cells were transferred to 12-well plates. The next day, cells were washed into DMEM+10% tetracycline-free FBS (Clontech) for 4 h. Transcription was halted with 1 μg ml^−1^ doxycycline, and timepoints were collected in Trizol (Life Technologies) after 30 min and at 3 h intervals thereafter. Northern blotting was performed as described using hexamer-labelled DNA probes against the full GFP sequence^22^, detected using a Storm 865 phosphorimager (GE Healthcare), and quantified on ImageStudio software (Li-Cor). Half-lives were determined from the best-fit line to semi-log plots of relative RNA abundances over time, and statistical significance was calculated by the ANCOVA analysis using Prism software (Graphpad).

### Data availability

The atomic coordinates and structural factors for MoMLV RT/eRF1 and MoMLV RNase H/eRF1-C have been deposited with the Protein Data Bank under accession codes 5DMQ and 5DMR, respectively. All other data are available from the corresponding authors on reasonable request.

## Additional information

**How to cite this article:** Tang, X. *et al*. Structural basis of suppression of host translation termination by Moloney Murine Leukemia Virus. *Nat. Commun.* 7:12070 doi: 10.1038/ncomms12070 (2016).

## Supplementary Material

Supplementary Information Supplementary Figures 1-5

## Figures and Tables

**Table 1:** Diffraction data collection and refinement statistics.

	MoMLV RT/eRF1	MoMLV RNase H/eRF1-C
*Data collection*		
Resolution limit (Å)	4.0	2.8
Space group	P3_2_21	C2
Cell parameters		
*a*/*b*/*c* (Å)	93.0/93.0/291.2	89.6/63.9/60.4
*α*/*β*/*γ* (°)	90.0/90.0/120.0	90.0/96.9/90.0
Unique reflections (N)	12,901 (3,557)	8,469 (1,229)
Total reflections (N)	82,665 (23,699)	57,658 (8,342)
Mean (*I*/*σ*)	6.9 (2.5)	23.6 (3.2)
Completeness (%)	99.7 (99.3)	99.9 (99.9)
CC(1/2)	0.99 (0.86)	1.0 (0.88)
*R*_merge_*	0.10 (0.70)	0.06 (0.62)
Redundancy	6.4 (6.7)	6.8 (6.8)
		
*Refinement statistics*		
Data range (Å)	96.7–4.0 (4.1–4.0)	60.0–2.8 (2.9–2.8)
Used reflections (N)	12,209 (849)	8,037 (614)
Protein residues	1,004	260
Protein atoms	7,878	2,041
*R*_work_†(%)	24.1	20.7
*R*_free_† (%)	29.5	28.0
r.m.s. deviation		
Bond length (Å)	0.010	0.012
Bond angles (°)	1.31	1.58
Ramachandran plot (% residues)		
Allowed	97.1	99.6
Generously allowed	1.5	0.4
Disallowed	1.4	0

Values in parentheses indicate the specific values in the highest resolution shell.

^*^R_merge_=∑|*I*_j_−<*I*>|/∑*I*_j_, where *I*_j_ is the intensity of an individual reflection, and <*I*> is the average intensity of that reflection.

^†^*R*_work_=∑||*F*_o_|−|*F*_c_||/∑|*F*_c_|, where *F*_o_ denotes the observed structure factor amplitude, and *F*_c_ denotes the structure factor amplitude calculated from the model.

^†^*R*_free_ is as for *R*_work_, but calculated with 5% of randomly chosen reflections omitted from the refinement.
