# Supplementary material for: Structural basis of suppression of host translation termination by Moloney Murine Leukemia Virus
Source: Nat Commun. 2016 Jun 22;7:12070. doi: 10.1038/ncomms12070 (PMC4917968; doi:10.1038/ncomms12070)
Supplement: Supplementary Information — Supplementary Figures 1-5 [file ncomms12070-s1.pdf]

Supplementary Figure 1

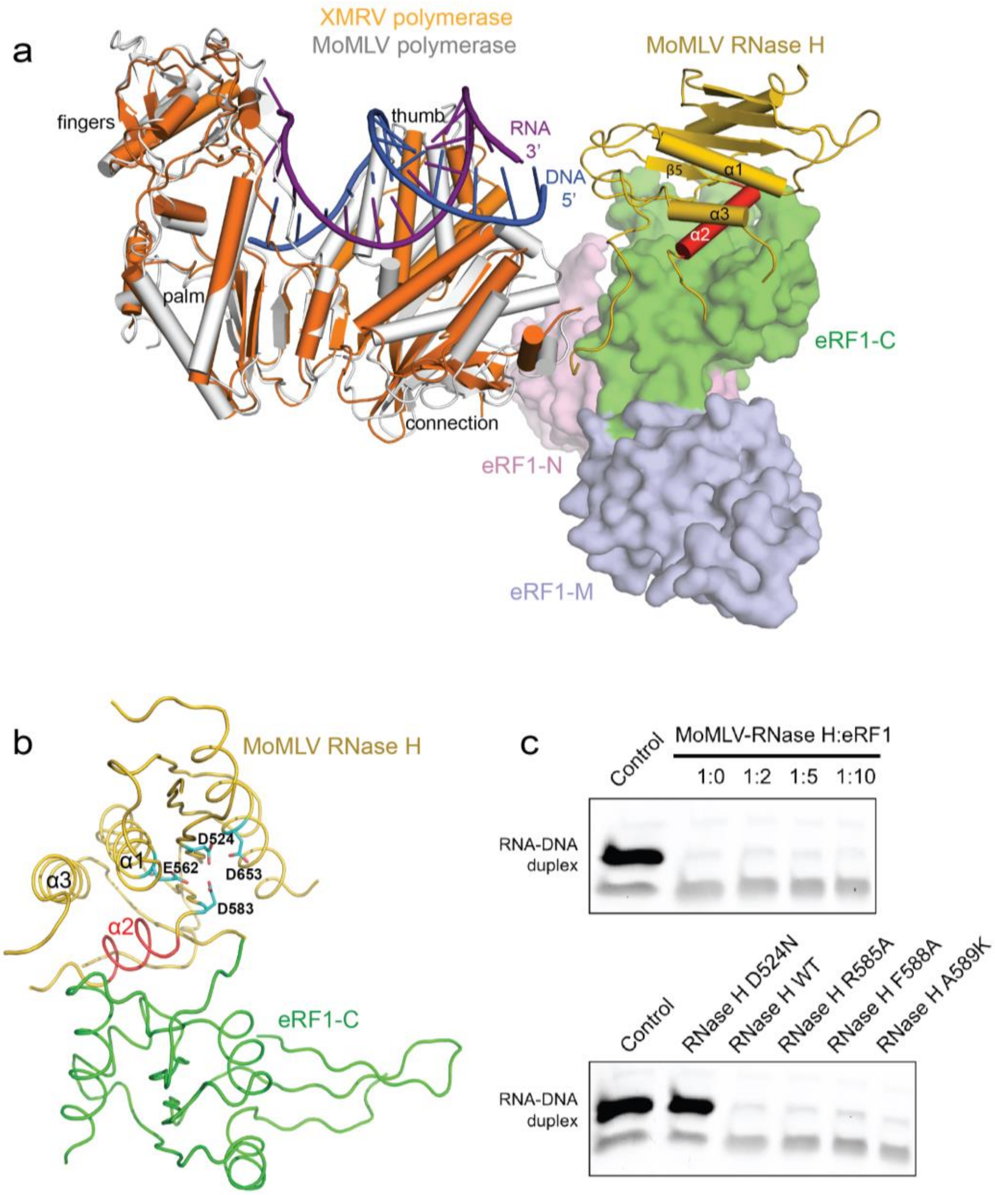

Supplementary Figure. 1. The binding of eRF1 to MoMLV RT does not affect RT's enzyme activities. (**a**) Superposition of the polymerase domains of MoMLV RT/eRF1 complex and XMRV RT/RNA/DNA ternary complex (4HKQ). The coloring scheme of MoMLV RT/eRF1 is as in Fig. 1. XMRV RT is colored in orange while the bound RNA and DNA in purple and blue respectively. The four subdomains of the polymerase domain are labeled. (**b**) The catalytic residues of MoMLV RNase H are distant from the bound eRF1. (**c**) RNase H activity of the MoMLV RNase H domain is not affected by its interaction with eRF1 (upper panel), and the mutations (R585A, F588A and A589K) did not affect their RNase H activities (lower panel).

Supplementary Figure 2

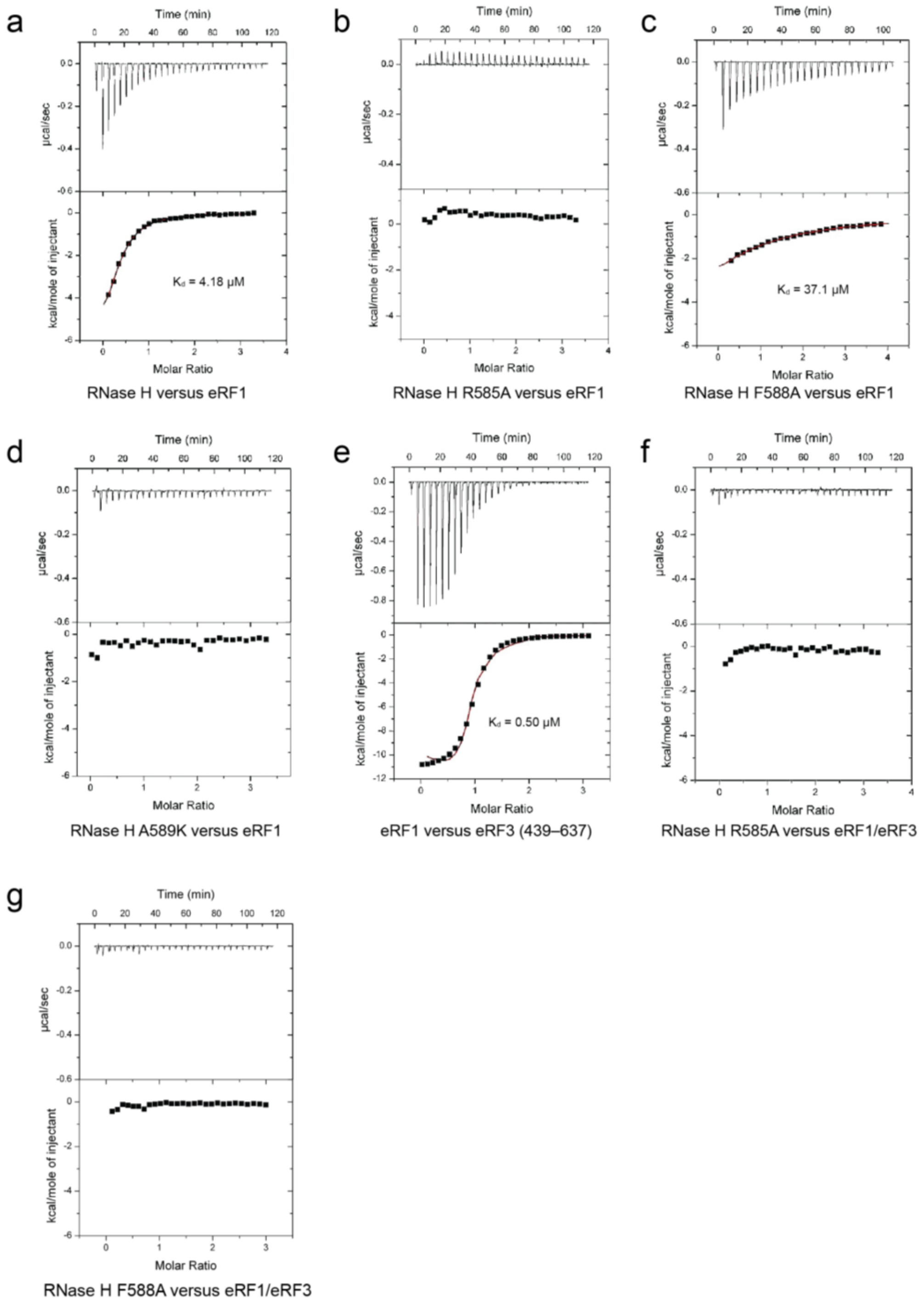

Supplementary Figure. 2. **Summary of isothermal titration calorimetry measurements.** (**a**) ITC titration of MoMLV RNase H and its variants (**b**) R585A, (**c**) F588A and (**d**) A589K to eRF1. (**e**) ITC titration of eRF1 to eRF3 (aa 439-637). (**f** and **g**) ITC titrations of MoMLV RNase H mutants R585A (f) and F588A (g) into the eRF1/eRF3 complex.

Supplementary Figure 3

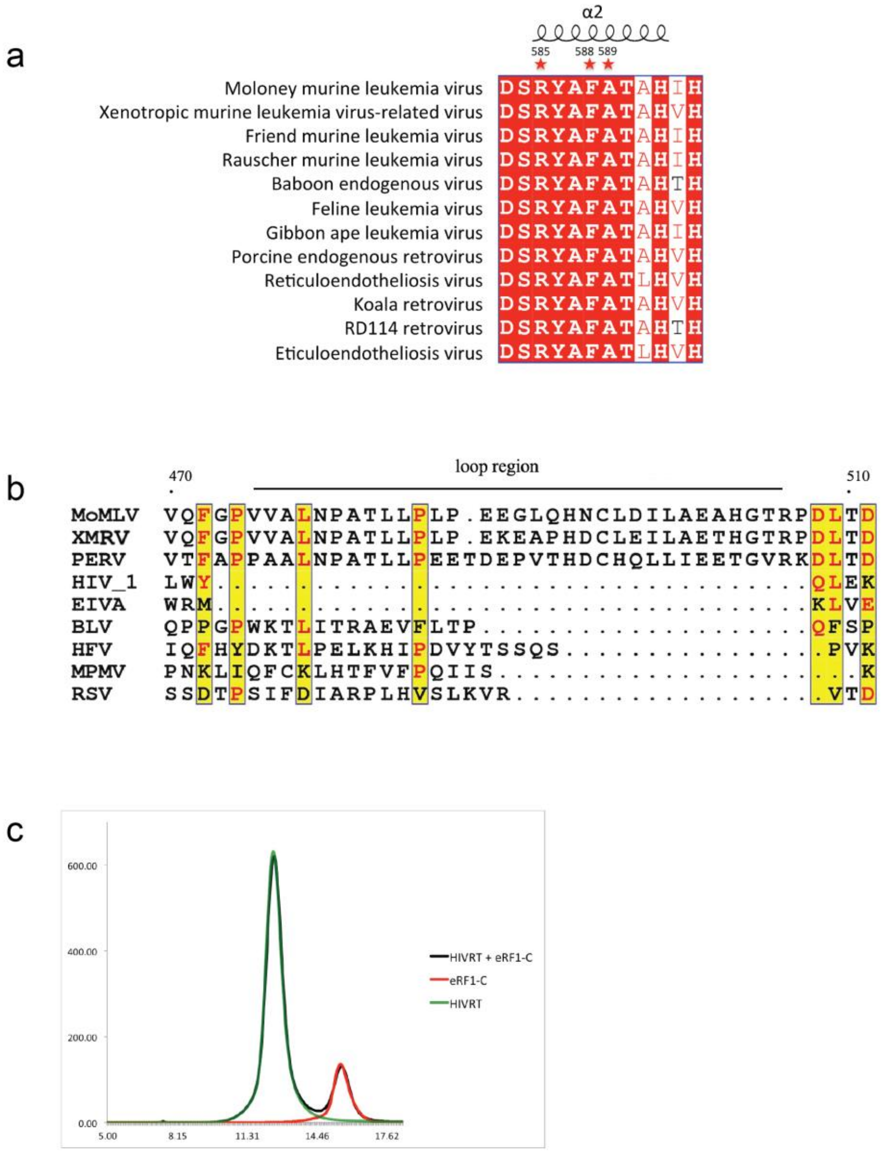

Supplementary Figure. 3. Retroviral RT. (**a**) Sequence alignment of the helix α2 from various gammaretrovirus. (**b**) Sequence alignment of the loop region between the connection subdomain and the RNase H domain for various retroviruses. (**c**) Size-exclusion chromatogram of HIV RT mixed with eRF1-C (black) at equimolar ratio overlaps with those of individual components, eRF1-C (red) and HIV RT (green), indicating there is no binding of HIV RT and eRF1-C domain.

Supplementary Figure 4

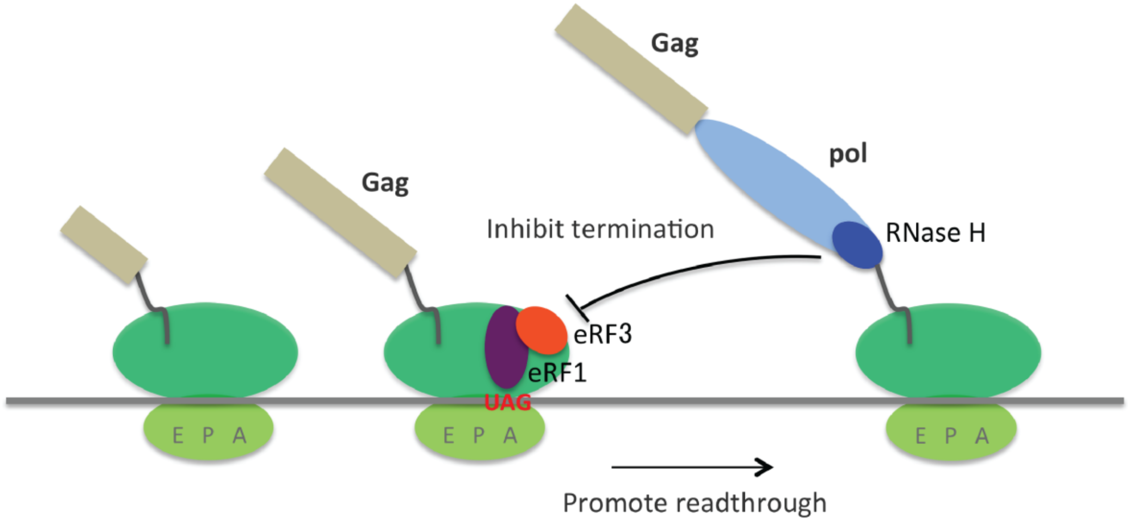

Supplementary Figure. 4. Model for the mechanism of RT RNase H domain to promote readthough in cis. The cartoon shows termination suppression activity may be strongest in *cis*, due to the newly synthesized MoMLV-RT RNase H polypeptide reaching back at the upstream termination codon on the same mRNA to interact with eRF1 and enhance readthrough.

Supplementary Figure 5

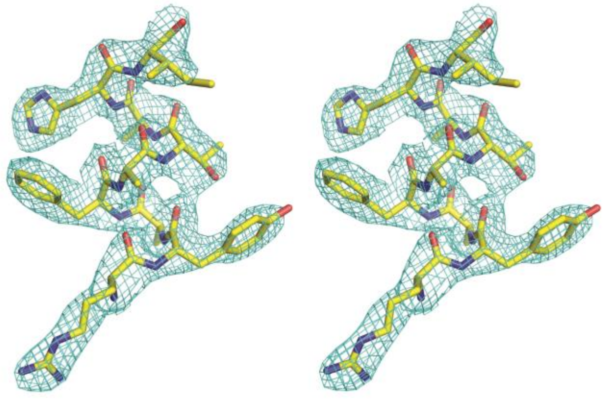

Supplementary Figure 5. Stereo view of a representative part of electron density map around the RNase H helix α2. The 2Fo−Fc electron density map is drawn at 1.5σ contour level. The atom colors are as follows: carbon, yellow; nitrogen, blue; oxygen, red.
